# Translation and validation of the Hebrew HITS screening tool for Intimate Partner Violence (IPV)

**DOI:** 10.1186/s12889-026-26480-8

**Published:** 2026-02-06

**Authors:** Daniel J. N. Weishut, Ruth Soffer-Elnekave, Anat Vass, Sara Zalcberg

**Affiliations:** 1 Department of Psychology, Jerusalem Multidisciplinary College, Jerusalem, Israel; 2 Department of Social Work, Augsburg University, Minneapolis, MN USA; 3 School of Social Work, Jerusalem Multidisciplinary College, Jerusalem, Israel; 4 The Charlotte B. and Jack J. Spitzer Department of Social Work, Ben-Gurion University of the Negev, Beer Sheva, Israel; 5 Shandong-Tel Aviv Joint Institute for Jewish and Israel Studies, Tel Aviv, Israel

**Keywords:** Intimate partner violence, IPV, Domestic violence, Screening tool, HITS, Hebrew, Social work students, Validation, Cross-cultural adaptation, Israel

## Abstract

**Background:**

Intimate partner violence (IPV) is a critical public health and human rights issue that remains underreported and often undetected, particularly in cultural contexts where stigma or taboo hinder disclosure. The Hurt, Insult, Threaten, Scream (HITS) screening tool is a brief, validated measure widely used in clinical and community settings for IPV detection (Sherin et al., Fam Med 30:508–12, 1998). However, a Hebrew-language version of the HITS tool has not previously existed.

**Methods:**

We translated and validated the HITS tool into Hebrew using a multi-step process: forward translation, expert panel review, back-translation, and cognitive appraisal with 18 diverse participants. This was followed by field testing with 219 Hebrew-speaking students and recent graduates of Social Work in Israel, of whom 144 were women (M = 27) and 75 were men (M = 31). The study obtained prior approval by the Institutional Review Board of the Jerusalem Multidisciplinary College (no. 2023 − 382).

**Results:**

The Hebrew HITS scale demonstrated acceptable to strong internal consistency (Cronbach’s α = 0.75; McDonald’s ω = 0.864) and good structural validity. Confirmatory factor analysis indicated an acceptable model fit across multiple indices. Gender differences emerged, with men reporting significantly higher IPV scores than women, while no significant differences were found across levels of religiosity.

**Conclusions:**

The Hebrew version of the HITS tool is a valid and reliable instrument for IPV screening in Hebrew-speaking populations and holds promise for use in clinical, academic, and community settings in Israel. Further research is needed to assess its use in more diverse and high-risk populations.

**Supplementary Information:**

The online version contains supplementary material available at 10.1186/s12889-026-26480-8.

## Background

Intimate partner violence (IPV) constitutes a major public health and human rights concern with profound psychological, physical, and social consequences. It affects individuals across all cultural, ethnic, and socioeconomic groups and has been linked to long-term health problems, reduced quality of life, and an increased burden on health and welfare systems [1, 2]. Despite increased awareness, IPV often remains underreported and undetected, particularly in communities where cultural taboos, stigma, or systemic barriers hinder disclosure. Timely identification through effective screening tools is therefore essential for prevention and intervention. This study addresses a key gap in IPV detection in Israel: the absence of a validated Hebrew-language screening tool appropriate for use in culturally diverse populations, including ultra-Orthodox communities.

The World Health Organization (WHO) defines IPV as “any behavior by a current or former male intimate partner within the context of marriage, cohabitation, or any other formal or informal union that causes physical, sexual, or psychological harm” ([3] p. 4). While acknowledging that women can also perpetrate IPV and that it occurs in same-sex relationships, the WHO emphasizes that IPV is most perpetrated by men against women and that it transcends national, cultural, and ethnic boundaries [3]. A review of 17 studies conducted during the COVID-19 pandemic—the period in which this study took place—identified factors such as depression, increased close contact, job loss, financial instability, lockdowns, addiction, control of household finances, technology, and quarantine as exacerbating the risk of violence toward women and children [4].

According to the WHO, 30% of women have experienced either physical and/or sexual intimate partner violence or non-partner sexual violence during their lifetime, with most of this violence perpetrated by intimate partners. Globally, about 27% of women aged 15–49 who have been in a relationship report experiencing some form of physical and/or sexual violence from their partner [5]. There is much less research on IPV toward men, an issue that is often silenced. However, recent studies have highlighted the barriers men face in disclosing IPV and seeking help, including stigma and traditional gender expectations [6–8] A meta-analysis of 30 studies reported that physical IPV toward men was 20%, psychological IPV was 44%, and sexual IPV was 7% [9].

IPV screening in Israel presents unique cultural and societal challenges, especially within the ultra-Orthodox Jewish community, where the discussion of sensitive issues like abuse and sexuality is taboo [10]. This barrier highlights the importance of culturally adaptable tools that maintain psychometric integrity while respecting community boundaries.

A nationally representative survey conducted in Israel using a stratified probability sample of over 2,500 households found that psychological aggression against women was slightly higher than in Western countries, while physical aggression rates were somewhat lower [11]. In 2017, it was estimated that approximately 200,000 women in Israel (out of a female population of roughly 4 million) were subjected to physical violence. However, due to insufficient data on the prevalence of other forms of violence—including sexual, economic, legal, and psychological—and the fact that only a quarter of victims report such crimes, the actual number of women affected by violence is likely significantly higher [12]. A recent study of over 2,000 Israeli adults found that 8.9% of women and 5.7% of men reported experiencing physical violence from their partner in the past year; 8.9% of women and 9.8% of men reported experiencing non-physical violence, and 12% of women and 9% of men reported experiencing sexual violence [13].

The vast diversity of Israeli society presents numerous challenges for policymakers and service providers in addressing IPV at all levels. In particular, there remains a lack of research on IPV within faith-based communities and the development of effective, culturally sensitive interventions for these communities [14, 15]. A study comparing IPV prevalence among three groups of women in Israel found an overall prevalence of about 40%, with Arab women reporting twice the rate of any IPV compared to immigrant and non-immigrant Jewish women (67%, 30%, and 27%, respectively). One-quarter of the participants—including Arab and non-immigrant Jewish women—self-identified as religious [16]. Some studies have indicated that high levels of religiosity and membership in collectivist patriarchal minority groups may contribute to a higher risk of IPV [16, 17] .

Estimates of IPV prevalence among young adults range from 10% to 20%, with some studies suggesting rates among college students as high as 50% [18, 19]. Specifically, they are at risk for dating violence [20–22], in which both young men and women are victims and perpetrators, often in the context of mutually violent relationships. College students are particularly vulnerable because many are in their first intimate relationships and may lack effective communication and relationship skills [23].

In Israel, Goussinsky and colleagues found that 20%–25% of college students experienced various forms of physical violence in their intimate relationships, and more than 10% reported physically attacking their partners [24]. As emerging adults, college students are in a life stage during which they develop relationship patterns and skills that influence future partnerships and marriages. The high occurrence of IPV at this stage presents a risk factor for experiencing violence in later adult relationships. The literature also suggests that university students who are exposed to or witness violence in their families show a higher tendency toward violence themselves and more negative behaviors compared to those who did not witness violence [25].

Universal screening for IPV, such as in emergency rooms or public clinics, has been found to increase the likelihood of identification beyond targeted screening approaches [26, 27] Screening can reduce victimization and improve health and wellbeing [28]; yet universal screening is still not widely implemented. Barriers include structural factors (e.g., lack of protocols, time constraints, insufficient referral systems) and attitudinal barriers [27, 28]. Thus, a brief, accessible screening tool is crucial for implementing IPV detection practices across health and social services.

### The current study

Recognizing the importance of addressing intimate partner violence (IPV), the first, second, and fourth authors—Social Work faculty members at a college with both secular and ultra-Orthodox campuses—organized a series of study days on the topic. As part of this initiative, they sought to map students’ experiences with IPV. A review of available Hebrew-language IPV instruments revealed that most were either unpublished or lacked psychometric data. The only validated Hebrew questionnaire located was the Revised Conflict Tactics Scales (CTS2) [29], a comprehensive tool commonly used in IPV research. However, we refrained from using the CTS2 due to institutional and cultural constraints as it includes explicit questions on sexual violence, to which religious authorities at the college objected.

As an alternative, the HITS (Hurt, Insult, Threaten, Scream) screening tool developed by Sherin et al. was selected [30]. HITS is a brief, self-report measure with four items that can be completed quickly in clinical or research contexts. It has been validated across a wide range of languages and cultural contexts, including Spanish [31], Arabic [32], Persian [33], Portuguese [34], and Turkish [35]. Despite this broad international use, no validated Hebrew version existed at the time of this study.

This absence represented a significant gap in IPV detection efforts, particularly given the high prevalence of IPV in Israel and the growing consensus about the importance of culturally appropriate screening tools in both clinical and public health settings [31, 36]. At the time of writing, Israel was experiencing war and internal displacement due to armed conflict [37]. Global research has demonstrated that IPV increases during wartime and humanitarian crises [38–41], underscoring the urgency of equipping health and welfare professionals with efficient IPV screening instruments adapted to local conditions.

Recent studies in public health have emphasized the importance of culturally adapted and efficient tools for IPV screening. For example, García and colleagues. validated a brief electronic control screener for young women in Spain [42], and Innab and others linked IPV to psychological distress among women in Saudi Arabia [43]. A recent meta-analysis highlighted that methodological differences in IPV measurement, including the use of brief tools such as HITS, influence prevalence estimates [44]. These findings underscore the need for validated IPV screening instruments adapted to local cultural and linguistic contexts.

The current study aimed to translate and validate a Hebrew version of the HITS tool using a multistep cultural and linguistic adaptation process in line with international best practices [45–47]. In addition to describing this process, we evaluated the psychometric properties of the resulting instrument among Hebrew-speaking Social Work students and recent graduates. This population was particularly relevant for three reasons. First, college students in Israel, especially in the ultra-Orthodox community, often marry young and may already be in long-term relationships. Second, students in the helping professions are themselves at risk for IPV and are also future implementers of IPV screening tools. Third, including students from both secular and religious campuses enabled an exploration of how gender and religiosity relate to IPV disclosure and measurement.

The study tested the internal consistency, factorial construct, and structural validity of the Hebrew HITS. We also explored score differences by gender and religiosity, two culturally salient variables in Israeli society that have been linked to IPV prevalence and help-seeking behavior in prior research [14, 16, 17]. The findings aim to inform efforts to integrate IPV screening into diverse educational, clinical, and community settings and to contribute to cross-cultural research on IPV measurement and prevention.

## Method

### Study design and ethics

**Fig. 1:**
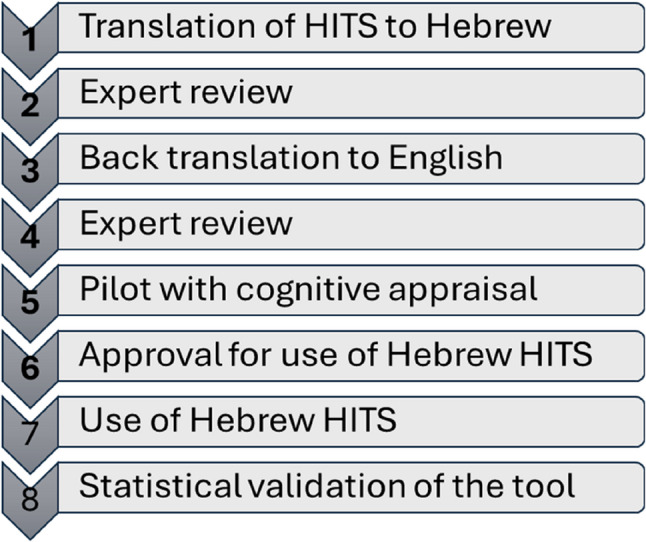
Steps in the translation and validation process of the Hebrew version of the HITS screening tool

The study focused on the translation to Hebrew and validation of the Hurt, Insult, Threaten, Scream (HITS) screening tool, which consists of four items scored on a 5-point Likert scale [30]. The study was approved by the Ethics Committee of the Jerusalem Multidisciplinary College (approval number 2023 − 382). The cross-cultural adaptation and validation process was rigorous, given the tool’s intended use in culturally diverse and religiously conservative populations, and included eight steps (see Fig. 1). The method employed was a multi-step approach for translating and validating scales, similar to those recommended by others [45, 46]. The first step involved forward translation of the psychometrically sound original English version into Hebrew. We obtained three translations of the English version into Hebrew: two by mental health professionals and one by a translator who was unaware of the translation’s purpose. The translations of the mental health professionals were highly similar, while the official translator’s translation was somewhat different.

The second step involved review by an expert committee, comprising two social workers, a clinical psychologist with experience in scale translation, and an anthropologist to provide insights into cultural and linguistic nuances, thereby enhancing the contextual appropriateness and interpretability of the scale in the ultra-Orthodox Jewish community. The review committee discussed the differences between the translations. To determine the optimal version, they consulted the translators to ascertain the rationale for the variations in wording. The official translator aimed to adhere closely to the original wording, while the mental health professionals sought to employ more colloquial and familiar language. Following this consultation, the committee opted to synthesize both versions, creating a third iteration that conveyed the original ideas while maintaining a close resemblance in wording.

The third step encompassed translating the Hebrew version back into English. This back-translation was conducted concurrently by a translator and a physician, unaware of the study’s purpose. Although not identical, both back-translations were highly similar to the original questionnaire.

In the fourth step, the expert panel meticulously reviewed the back-translation and agreed on a pre-final version. The expert panel encountered several linguistic and cultural challenges during the translation process. First, there were two viable translations for the word “scream” (tzo’ek and tzoreah); the more literal translation was selected for its back-translation accuracy. Second, the idiom “talk down to” had multiple Hebrew equivalents (yored al, mashpil, medaber behitnassut), and the phrase that best approximated “patronize” in back-translation was chosen. Third, the original HITS response scale—1-never, 2-rarely, 3-sometimes, 4-fairly often, and 5-frequently—was adapted to conform to standard Hebrew questionnaire phrasing. The Hebrew version used 1-never, 2-rarely, 3-sometimes, 4-often, and 5-very often, to enhance clarity and maintain conceptual equivalence. These decisions reflect the expert panel’s aim to balance linguistic fidelity with cultural and functional appropriateness for the target population.

The fifth step involved cognitive appraisal of the pre-final version. The expert panel recruited 18 adult subjects (10 women and 8 men) who completed the pre-final questionnaire and provided feedback on the questions. The subjects were deliberately diverse in terms of age (ranging from the early twenties to the mid-sixties), profession (students, academics, and non-academics), and religious background (ultra-Orthodox, conservative, and secular). In their reports, one subject noted a technical issue with completing the form on their cellular phone, two provided suggestions regarding wording, and four found the questionnaire confronting or alluded to potential reluctance among prospective subjects to complete the form due to its sensitive content. However, all subjects affirmed that the questionnaire was precise in its phrasing and that the intent of each item was clear and unambiguous.

The sixth step entailed reviewing the subjects’ feedback and approving a final Hebrew version of the tool. As no comments necessitated adaptation of the questionnaire, this version was retained as the final Hebrew tool (see Appendix). The subsequent two steps, concerning the utilization and statistical validation of the Hebrew HITS screening tool, will be explained below.

### Participants

We utilized a convenience sample of students and recent graduates of Social Work at a local college. The Bachelor of Social Work program spans three years and typically caters to students aged 20–30. The college comprises two distinct campuses: one serving students from all sectors of Israeli society, with approximately 70 students per cohort, and the other serving the Jewish ultra-Orthodox community, with approximately 50 students per cohort.

A total of 304 participants took part in the study. Participants who were in an intimate relationship at the time of data collection were asked to complete the HITS questionnaire; 229 met this criterion and completed the measure. Data from ten non-Jewish participants were excluded because their mother tongue was Arabic, whereas the present study focuses on the Hebrew version of the HITS; a validated Arabic version of the scale already exists [32]. Approximately 27% of cases contained missing data across the study variables. Missing data patterns were examined prior to analysis. Little’s MCAR test [48] yielded a non-significant result, χ²(3) = 6.34, *p* = 0.096, indicating no evidence that the data deviated from a missing completely at random (MCAR) mechanism. Inspection of missing data patterns revealed a single missingness pattern, suggesting that missingness was uniform rather than scattered across variables. Given these findings, analyses were conducted using complete-case analysis. The final analytic sample, following listwise deletion, consisted of *N* = 219 participants.

Of the 219 remaining subjects (144 women and 75 men), the vast majority (87%) were undergraduate Social Work students at the specified college, evenly distributed across the three cohorts, and 13% had recently graduated from the same institution. 66% of the subjects were female, 71% were married, and 2% were divorced. Most participants identified as religious: 58% were ultra-Orthodox, 19% were Religious-Nationalist (Modern Orthodox), 9% identified as traditional Jews, and 14% as secular Jews (two participants didn’t disclose their religiosity). The mean age of the subjects was 28, which is higher than in other countries. Israeli students study at a later age than their counterparts elsewhere, particularly ultra-Orthodox Jews who marry and start families before pursuing higher education, and Religious-Nationalist men and non-Orthodox women and men first serve in the military. These characteristics distinguish Israeli college students and mirror the unique demographics of the local college.

### Data collection

Data were collected prior to a series of study days in early 2023. By that time, all COVID-19 restrictions in Israel—mask mandates, social distancing requirements, and isolation orders—had been lifted. While the lingering psychosocial effects of the pandemic cannot be ruled out, the survey was conducted in a post-pandemic context and does not reflect responses under active containment measures. Ahead of the study days, all students and recent graduates were invited to participate through the faculty’s administrative office and were asked to complete an online questionnaire on intimate partner violence. Most consented to participate in the study, which was voluntary and offered no incentives. The HITS questionnaire was part of a longer questionnaire and was distributed only to participants currently in intimate relationships. The online survey also included a short demographic questionnaire, covering age, gender, marital status, religious affiliation (pre-defined), study year, and campus affiliation. Contact information of the researchers and services for treating intimate partner violence were presented at the end of the questionnaire in case of distress following participation in the study.

### Data analysis

Data analysis was performed using RStudio, a free and open-source integrated development environment (IDE) for R, a free software environment for statistical computing and graphics (R Version 4.4.1; RStudio 2023.06.1; main packages used: Lavaan and bruceR [49, 50]). Confirmatory factor analysis (CFA) was conducted using maximum likelihood estimation. Model fit was evaluated using the indices CFI, TLI, NFI, SRMR, and RMSEA, based on standard cutoffs.

## Results

### Descriptives, bivariate statistics, and mean comparison

The finalized Hebrew version of the HITS, shaped through expert panel review and cognitive appraisal detailed previously, was administered to 219 participants. The results below summarize its reliability, gender and religiosity-based comparisons, and factorial structure. We analyzed the responses of 219 subjects who completed the HITS questionnaire. On a scale of 4 to 20, the mean HITS score was 4.54 (SD 1.45; MIN 4, MAX 15), with 55 (25%) of subjects reporting any form of IPV. Three subjects (1.4%), two male and one female, scored over 10.5, the threshold differentiating between clinical and non-clinical populations [30] (see Table 1).

**Table 1:** Participant demographics and HITS score distributions

Demographic Variable	Category	*N*	%	Mean Age	Mean HITS Score (SD)
Gender	Female	144	66%	27	4.38 (1.07)
	Male	75	34%	31	4.85 (1.96)
Marital Status	Single	60	27%	24	4.19 (0.51)
	Married	155	71%	29	4.57 (1.33)
	Divorced	4	2%	33	6.25 (4.50)
Religiosity	Ultra-Orthodox	126	58%	30	4.67 (1.77)
	Religious-Nationalist	41	19%	25	4.27 (0.92)
	Secular/Traditional	50	23%	27	4.48 (0.76)
Total		219	100%	28	4.54 (1.45)

The HITS score ranges from 4 to 20

Both parametric and non-parametric analyses were conducted to examine potential gender differences in HITS scores. Descriptive statistics indicated that males (M = 4.85, SD = 1.96, *N* = 75) had higher mean HITS scores than females (M = 4.38, SD = 1.07, *N* = 144). A one-way ANOVA showed a significant effect of gender on HITS scores, F(1,217) = 5.315, *p* < 0.05, with a small effect size (η2 = 0.024). However, Levene’s test indicated that the assumption of homogeneity of variances was violated (*p* < 0.05), warranting further investigation using robust and non-parametric tests. A Welch’s ANOVA, which accounts for unequal variances, confirmed a significant gender effect, F(1,217) = 19.83, *p* < 0.001. To further validate this finding, a Mann-Whitney U test was conducted. The results indicated a statistically significant difference in the distribution of HITS scores between genders (U = 6275.00, z = 2.589, *p* < 0.010), with males (mean rank = 121.67) scoring higher than females (mean rank = 103.92) (see Table 1).

Descriptive statistics of Religiosity revealed the following HITS scores: secular/traditional, combined to decrease differences in group size (M = 4.48, SD = 0.76, *N* = 50), religious-nationalist (M = 4.27, SD = 0.92, *N* = 41), and ultra-Orthodox (M = 4.67, SD = 1.77, *N* = 126). A one-way ANOVA showed no statistically significant difference in HITS scores across the religious groups, F(2,214) = 1.233, *p* > 0.1. Welch’s ANOVA confirmed the non-significance of the religiosity-based differences (F(2,152.51) = 1.753, *p* > 0.1). Post-hoc comparisons using Tukey’s HSD and Bonferroni corrections showed no significant pairwise differences between any of the religious groups (*p* > 0.05).

### Confirmatory factor analysis

A Confirmatory Factor Analysis (CFA) was conducted to examine the structural validity and internal consistency of the total HITS score as it pertains to its components. The model included four variables relating to spousal IPV: Hurt (physical aggression), Insult, Threaten, and Scream. The accepted threshold number of observations needed for CFA is estimated at *N* = 200 [51]. Additionally, sources usually require a ratio of at least 10:1 observations for each observed variable (with some requiring a 20:1 ratio) [51, 52]. As our CFA consists of only 4 observed variables, the sample size of 219 satisfies both requirements and is considered satisfactory.

The chi-square test of the user model yielded a test statistic of 7.610 with 2 degrees of freedom, resulting in a significant p-value of 0.022. The chi-square test rendered a test statistic of 417.912 with 6 degrees of freedom for the baseline model. This model was highly significant, with a p-value of 0.000. Several fit indices were examined to compare the goodness of fit between the user and baseline models. The user model’s Comparative Fit Index (CFI) was 0.986, indicating a good fit to the data. The Tucker-Lewis Index (TLI) was found to be 0.959, and the Normed Fit Index (NFI) was 0.982, both supporting the adequacy of the user model in comparison to the baseline.

The log-likelihood for the user model (H0) was − 92.527, while for the unrestricted model (H1) it was − 88.722. The Akaike Information Criterion (AIC) was calculated as 201.054, and the Bayesian Information Criterion (BIC) as 228.167. The Sample-size adjusted Bayesian (SABIC) was noted as 202.815. The RMSEA [52] for the user model was 0.113. The 90% confidence interval for this statistic ranged from 0.036 (lower limit) to 0.203 (upper limit). The SRMR, another measure of goodness of fit, for the user model was found to be 0.023, indicating a close fit of the model to the observed data. A one-factor confirmatory factor analysis was conducted using the robust Maximum-Likelihood estimator. The model demonstrated good fit to the data, robust χ²(2) = 1.32, *p* = 0.52, with excellent incremental and absolute fit indices (robust CFI = 1.00, robust TLI ≈ 1.00, SRMR = 0.023). Although the non-robust RMSEA was elevated (0.113), robust RMSEA was 0.00. Given the very low degrees of freedom (df = 2) and the known instability of RMSEA in such models, e.g., [53], model fit evaluation emphasized robust incremental fit indices and SRMR. In conclusion, the user model provided an acceptable fit to the data, as indicated by multiple fit indices, and was significantly different from the baseline model.

**Fig. 2:**
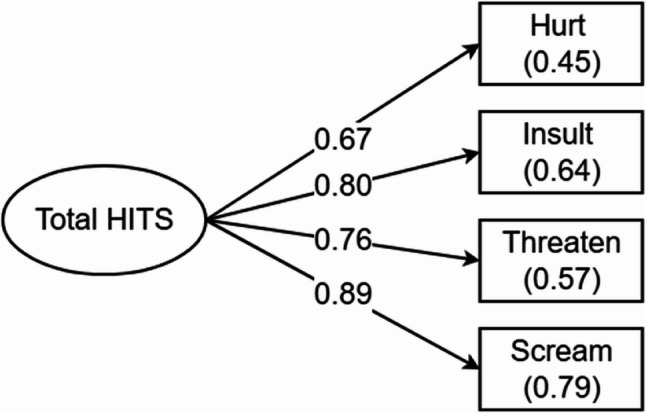
Confirmatory factor analysis of individual items on HITS scale with total HITS score. Note. The numbers along the lines are the factor loadings, all *p* < 0.001. The numbers in brackets pertain to the residual (explained) variance (R^2^) for each factor

The factors contributing to the total HITS score showed strong and statistically significant relationships with the observed variables (*p* < 0.001 for all loadings) (see Table 2). The strongest loading was for Scream (standardized loading = 0.89), followed by Insult (0.80), Threaten (0.76), and Hurt (0.67). The total HITS score explained 79% of the variance in screaming, 64% in insulting behavior, 57% in threats, and 45% in physical aggression (see Fig. 2). Overall, the CFA establishes the HITS scale’s structural validity and internal consistency, confirming that IPV-related behaviors cluster into a coherent latent construct.

**Table 2:** Confirmatory factor analysis and reliability measures of HITS

Variable	Factor loading	R2	Cronbach’s α	McDonald’s ω
Hurt	0.68	0.45	0.75	0.86
Insult	0.80	0.64		
Threaten	0.76	0.57		
Scream	0.89	0.79		
χ2(2) = 7.61, *p* = 0.02				
RMSEA = 0.113				
CFI = 0.986				
TLI = 0.959				
NFI = 0.982				
AIC = 201.054				
BIC = 228.167				

In addition to structural validity, the Hebrew HITS scale’s internal consistency was examined. Cronbach’s alpha was 0.75, and McDonald’s omega was 0.864, indicating acceptable to strong reliability for a brief screening instrument. Item-total correlations ranged from 0.638 (physical aggression) to 0.762 (scream), and the removal of any single item would have reduced overall reliability (e.g., α = 0.554 if scream were excluded), supporting the inclusion of all four items.

## Discussion

The results of our study show that the Hebrew version of the HITS screening tool is reliable and valid. Confirmatory factor analysis supported the scale’s structural coherence, with statistically significant item loadings. In addition to structural validity, internal consistency indices - Cronbach’s alpha and McDonald’s omega - supported the scale’s reliability, and item-total correlations confirmed that each item contributed meaningfully to the total score. These findings suggest that, while the items are conceptually related—as expected in a brief IPV screening tool—they each capture distinct dimensions of partner violence and, together, form a coherent unidimensional scale.

While moderate inter-item correlations were expected, given the conceptual proximity of IPV behaviors, the absence of excessively high correlations or distorted model fit statistics suggested that multicollinearity was not a problem. Alternative modeling strategies were considered, but the original four-item unidimensional structure was retained in accordance with the design and purpose of the original HITS tool. Although translation decisions were described in the Method section, it is worth noting that the process underscored the complexity of culturally adapting IPV screening items. Careful linguistic choices were required to preserve the original tool’s intent while ensuring clarity and usability in Hebrew.

The mean HITS score in the present study was 4.54 (on a range of 4 to 20), which is substantially lower than the mean of 6.13 for a non-clinical population reported by its developer [30]. Furthermore, the original research suggested a cutoff score of 10.5 to differentiate between self-identified victims and office workers. In the current study, only three subjects (1.4%), two men and one woman, scored above this threshold. The lower incidence of IPV in this sample may partly reflect increased awareness and sensitivity to IPV, possibly influenced by global public health efforts, including those led by the World Health Organization [3, 5]. However, as discussed in the limitations, underreporting may also have been influenced by stigma, religious or cultural norms, and self-selection bias. These factors should be considered when interpreting the prevalence findings. Noteworthy is that Sherin et al. emphasized the importance of investigating the possibility of intimate partner violence whenever a concern arises [30], irrespective of the cutoff score, as IPV could still be an issue. Fifty-five (25%) of the students in the present study reported some violence by their partner, which permitted analysis of the reliability and validity of the Hebrew version of the HITS screening tool.

The psychometric findings of the Hebrew HITS are broadly consistent with validation studies conducted in other settings. In the original development study, Sherin et al. [30] reported good internal consistency for the four-item scale (Cronbach’s α = 0.80). In a US clinical sample, Chen et al. found Cronbach’s alpha values of 0.76 for the English version and 0.71 for the Spanish version when administered before other questionnaires [31]. The Turkish validation reported high internal consistency (α = 0.89) and a clear one-factor structure [35]. In the Persian adaptation, item-level Cronbach’s alpha coefficients were extremely high (α = 0.977–0.994), indicating strong reliability for each item [33]. In our study, the Hebrew version demonstrated internal consistency within this broader international range (α = 0.75; ω = 0.86) and replicated the unidimensional structure consistently reported across adaptations. Taken together, these findings show that despite variability in reliability across studies, the HITS scale maintains a stable single-factor structure across cultures—even between linguistically distant languages such as English and Hebrew—and demonstrates acceptable internal consistency in the present adaptation.

While there was no significant difference in HITS scores across levels of religiosity, a small but significant gender difference emerged, suggesting that men may experience a bit more violence than do women. Although this might suggest greater IPV exposure among men in this sample, the finding may instead reflect the unique characteristics of male social work students. Social Work is a predominantly female profession, with 90% of Israeli social workers being women [54], yet only 66% of participants in this study were women due to the inclusion of students from the ultra-Orthodox all-male campus. Prior research has noted that men in caring professions often embody non-traditional masculinities and may be more emotionally expressive [55]. Indeed, Segev and Lander found that Israeli male social workers often strive to actualize their emotional selves, which may facilitate greater disclosure of intimate experiences [56]. This may help explain their higher reported IPV scores, despite the fact that, in general, men are less likely to report IPV victimization due to stigma and traditional gender norms [6–8]. As argued, male social workers are not demographically representative of men more broadly, which further limits the generalizability of the findings [57]. Nonetheless, findings on gender differences in IPV among college students remain inconsistent [58], underscoring the need for research specifically on how male social workers perceive and report IPV compared to their female counterparts.

Regarding the statistical tests, all results indicated good fit except for RMSEA. There are at least two possible reasons that RMSEA exceeded conventional thresholds. RMSEA assumes that the data has a normal distribution [59]. In our case, this is not the situation. Most subjects reported no violence, whereas only very few reported physical violence. If the data is not normally distributed, this can lead to inflated RMSEA values. Another possible reason is the limited degrees of freedom [60]. RMSEA penalizes model complexity through the degrees of freedom—when the degrees of freedom are small, even a modest chi-square value may yield a relatively high RMSEA value, potentially overstating the degree of model misfit. In our study, there were only 2 degrees of freedom, making this a distinct possibility. Together, these considerations invite future research to examine the explanatory processes suggested here, using designs that extend beyond the structural validation focus of the present study.

Translating and validating the HITS (Hurt, Insult, Threaten, Scream) screening tool for assessing IPV among Israel’s Hebrew-speaking population is crucial for several reasons. Primarily, it ensures cultural and linguistic relevance, maintaining the tool’s effectiveness in the Israeli context. This process extends beyond mere translation, taking into account cultural nuances to preserve the tool’s intended meaning. Validation is essential to confirm the tool’s psychometric properties in the new cultural setting, ensuring reliable data collection for research and intervention purposes [61]. An adequately translated and validated tool enables meaningful cross-cultural comparisons, thereby contributing to the international standardization of quality-of-life measures [62]. Moreover, it enables early identification of students at risk, facilitating timely interventions and support services. The translation and validation process also ensures cultural sensitivity, increasing the likelihood of accurate responses and reducing misinterpretation due to cultural differences [63].

The translation and validation process often involves collaboration with local experts, enhancing the cultural competence of researchers and practitioners in the field [64]. Ethically, using a properly translated and validated tool demonstrates respect for linguistic and cultural diversity [65]. It also contributes to the global body of knowledge on family violence assessment tools and their cross-cultural applicability, potentially informing future research and tool development in other cultural contexts [66]. Ultimately, by translating and validating the HITS tool for use with Israeli students, researchers can ensure they use a culturally appropriate, reliable, and valid instrument to assess IPV, which is crucial for conducting meaningful research, developing effective interventions, and working towards reducing family violence in Israel.

Although this study focused on translating and validating the HITS tool for use among Hebrew speakers, additional research should assess its performance in more diverse and higher-risk populations, as IPV experiences and disclosure patterns may vary significantly, for example, among recent immigrants, LGBTQ+ individuals, non-Jewish minorities, or those in shelter or clinical settings. Evaluating the tool’s cultural sensitivity and psychometric performance across such populations will be essential for ensuring its broader utility in both public health and family practice settings.

From a practical perspective, the Hebrew HITS provides health, welfare, and educational professionals in Israel with a brief, culturally accessible tool for early detection of IPV. Its brevity allows routine screening in settings with time or resource constraints, such as primary care, emergency rooms, women’s health clinics, and campus counseling centers. The tool may be particularly useful in ultra-Orthodox settings where more extensive IPV instruments are considered intrusive or culturally inappropriate. Incorporating the Hebrew HITS into routine assessment protocols can enhance the likelihood of identifying individuals at risk, improving referral pathways to support services, and potentially reducing the health and psychosocial burden associated with IPV.

### Strengths and limitations

This study has several methodological strengths. It employed a rigorous multi-step translation and cross-cultural adaptation process in accordance with established international guidelines. The sample included students from both secular and ultra-Orthodox campuses, enabling examination across diverse sociocultural groups rarely represented in IPV research. The use of multiple indices of reliability and CFA provides robust evidence for the structural validity of the Hebrew HITS. These strengths collectively enhance the cultural relevance and psychometric soundness of the translated tool.

Several limitations in this study should be acknowledged and could possibly support alternative explanatory hypotheses. First, although the primary focus of this study was the translation and cultural adaptation of a screening tool, it is necessary to address the representativeness of the sample. The study relied on a convenience sample of Social Work students from a single institution, which may limit the generalizability of the findings to other populations and professions. Due to their professional identity, Social Work students may possess a heightened awareness of IPV-related issues, potentially leading to disclosure patterns characterized by more cautious or informed responses than the general public. Another concern is the sample composition. The sample included a high proportion of ultra-Orthodox students (56%), whose cultural and religious norms may shape both experiences of and responses to IPV. Although men accounted for 34% of the sample—a relatively high proportion for this field—they might not represent the wider male population in Israel. Furthermore, since participation was voluntary and initiated through administrative outreach, there is a risk of self-selection bias, whereby individuals more aware of or affected by IPV may have been more inclined to participate. As the study focused exclusively on Hebrew-speaking participants, the findings do not extend to Arabic-speaking populations, for whom a validated Arabic version of the HITS tool already exists.

Another limitation is the non-normal distribution of responses: most participants reported no IPV, and only three reported IPV above the clinical threshold. The relatively small sample size also limited the scope of statistical analyses that could be conducted. For instance, multivariate analyses to explore underlying factors influencing IPV reporting were limited by insufficient variability in the data. Additionally, multicollinearity among questionnaire items may have affected some model fit indices, particularly the RMSEA, underscoring the complexity of analyzing self-reported IPV data.

The Hebrew version of the HITS was not validated against the Revised Conflict Tactics Scales (CTS2) [29], which remains the most widely used instrument for assessing IPV in Israel. While such a comparison would have strengthened convergent validity, it was not feasible in this study as it includes items on sexual violence that were deemed culturally inappropriate by the religious authorities of the institution. Although this concern served as the incentive for translating HITS, it prevented the inclusion of the CTS2 as a benchmark instrument.

Finally, a limitation of the HITS questionnaire, in any language and not specifically to this study, is its reliance on self-report for data collection. IPV is a highly sensitive topic, and participants may have underreported experiences of abuse due to social desirability bias, fear of stigma, or discomfort in disclosing personal information in an academic context. Although steps were taken to ensure privacy and anonymity, these concerns may still have influenced participants’ responses.

## Conclusion

This article detailed the translation and validation of the HITS screening tool for identifying Intimate Partner Violence (IPV) among Hebrew-speaking populations, with an emphasis on its utility in public health and clinical contexts. The translation process addressed linguistic and cultural challenges. Through a multi-step methodology—including expert review, cognitive appraisal, and psychometric testing—the Hebrew version of the HITS demonstrated acceptable internal consistency and structural validity within a sample of Social Work students. Because of its brevity and ease of administration, the Hebrew HITS can be readily integrated into routine screening practices in primary care clinics, women’s health services, emergency departments, and other health or clinical environments.

While the findings support the Hebrew HITS as a reliable instrument for IPV screening, several limitations should be noted. The small number of participants scoring above the clinical threshold reflects the characteristics of the student sample but limits the ability to assess the tool’s clinical sensitivity in higher-risk populations. Additionally, multicollinearity among items and a non-normal response distribution suggest the need for further psychometric refinement. Moreover, the voluntary nature of participation and the religious-academic setting may have introduced self-selection bias, thereby reducing the generalizability of the findings. Future research should evaluate the Hebrew HITS in more diverse and clinical populations, including those at higher risk of IPV, compare its performance with that of established instruments, such as the Revised Conflict Tactics Scales, and establish convergent and discriminant validity. Mixed-method studies, including qualitative interviews, could further contextualize responses and enhance cultural sensitivity. Methodological improvements—such as testing alternative factor models—may also improve model fit and clarity.

Despite these limitations, the study presents a critical first step toward developing a brief, easy-to-administer IPV screening tool accessible to Hebrew-speaking populations and that accommodates sensitivities within ultra-Orthodox communities. The Hebrew HITS holds promise for supporting early detection and intervention across health, welfare, and community services, particularly if validated in broader populations. Like other screening tools, the HITS scale should not be the sole measure of IPV but must be used in conjunction with indirect questions or anonymous methods, especially with populations that are less likely to disclose information directly.

## Supplementary Information

Supplementary Material 1.

## Data Availability

Anonymized datasets related to the psychometric analyses and the translation process are available from the corresponding author upon reasonable request. Due to the sensitive nature of IPV data and ethical restrictions associated with participant confidentiality, the full raw dataset cannot be made publicly available.
