# Supplementary material for: Translation and validation of the Hebrew HITS screening tool for Intimate Partner Violence (IPV)
Source: BMC Public Health. 2026 Feb 6;26:848. doi: 10.1186/s12889-026-26480-8 (PMC12977737; doi:10.1186/s12889-026-26480-8)
Supplement: Supplementary file 1 — Supplementary Material 1. [file 12889_2026_26480_MOESM1_ESM.docx]

**Translation and Validation of the Hebrew HITS Screening Tool for Intimate Partner Violence (IPV)**

Daniel J.N. Weishut, Ruth Soffer Elnekave, Anat Vass & Sara Zalcberg

Correspondence: [danielwe@jmc.ac.il](mailto:danielwe@jmc.ac.il)

**שאלון אלימות זוגית (HITS)**

באיזו תדירות בן.בת הזוג שלך פוגע.ת בך פיזית?

באיזו תדירות בן.בת הזוג שלך מעליב.ה אותך או מדבר.ת אליך בהתנשאות?

באיזו תדירות בן.בת הזוג שלך מאיים.ת לפגוע בך?

באיזו תדירות בן.בת הזוג שלך צורח.ת עליך או מקלל.ת אותך?

1. אף פעם
2. לעיתים רחוקות
3. לפעמים
4. לעיתים קרובות
5. לעתים קרובות מאד

תרגום השאלון:

דניאל ג'.נ. ווייסהוט, רות סופר-אלנקוה, ענת וואש & שרה זלצברג (2023), המכללה האקדמית הדסה.

מקור השאלון:

Sherin, K., Sinacore, J., Li, X. Q., Zitter, R. E., & Shakil, A. (1998). HITS: A short domestic violence screening tool for use in a family practice setting. *Family Medicine*, 30(7), 508–512.
